# Mechanistic Study of the Phytocompound, 2- *β* -D-Glucopyranosyloxy-1-hydroxytrideca-5,7,9,11-tetrayne in Human T-Cell Acute Lymphocytic Leukemia Cells by Using Combined Differential Proteomics and Bioinformatics Approaches

**DOI:** 10.1155/2015/475610

**Published:** 2015-10-18

**Authors:** Jeng-Yuan Shiau, Shu-Yi Yin, Shu-Lin Chang, Yi-Jou Hsu, Kai-Wei Chen, Tien-Fen Kuo, Ching-Shan Feng, Ning-Sun Yang, Lie-Fen Shyur, Wen-Chin Yang, Tuan-Nan Wen

**Affiliations:** ^1^Agricultural Biotechnology Research Center, Academia Sinica, No. 128, Sec. 2, Academia Road, Nankang, Taipei, Taiwan; ^2^Institute of Biotechnology, National Taiwan University, 4F, No. 81, Chang-Xing Street, Taipei, Taiwan; ^3^Department of Life Sciences, National Chung Hsing University, Life Sciences Building 2F-7F, 250 Kuo Kuang Road, Taichung, Taiwan; ^4^Institute of Plant and Microbial Biology, Academia Sinica, Taiwan

## Abstract

*Bidens pilosa*, a medicinal herb worldwide, is rich in bioactive polyynes. In this study, by using high resolution 2-dimensional gel electrophoresis coupled with mass spectrometry analysis, as many as 2000 protein spots could be detected and those whose expression was specifically up- or downregulated in Jurkat T cells responsive to the treatment with 2-*β*-D-glucopyranosyloxy-1-hydroxytrideca-5,7,9,11-tetrayne (GHTT) can be identified. GHTT treatment can upregulate thirteen proteins involved in signal transduction, detoxification, metabolism, energy pathways, and channel transport in Jurkat cells. Nine proteins, that is, thioredoxin-like proteins, BH3 interacting domain death agonist (BID protein involving apoptosis), methylcrotonoyl-CoA carboxylase beta chain, and NADH-ubiquinone oxidoreductase, were downregulated in GHTT-treated Jurkat cells. Further, bioinformatics tool, Ingenuity software, was used to predict signaling pathways based on the data obtained from the differential proteomics approach. Two matched pathways, relevant to mitochondrial dysfunction and apoptosis, in Jurkat cells were inferred from the proteomics data. Biochemical analysis further verified both pathways involving GHTT in Jurkat cells. These findings do not merely prove the feasibility of combining proteomics and bioinformatics methods to identify cellular proteins as key players in response to the phytocompound in Jurkat cells but also establish the pathways of the proteins as the potential therapeutic targets of leukemia.

## 1. Introduction

The omics technologies are being increasingly utilized as the systems biology approach to study diseases and drug discovery [1]. For instance, proteomics research using a combination of techniques, including high-resolution 2-dimensional electrophoresis analysis and mass spectrometry (2DE/MS) for protein identification, has become a powerful approach in the rapid identification and validation of protein expression profiles, posttranslation modifications, and new protein targets. The proteomics approach can also lead to identifying potential biomarkers and elucidate cross-talk molecules among signal pathways in human cancer diseases [2]. Bioinformatics is emerging as an indispensable tool to process and analyze the big data generated from omics technologies. The development of bioinformatics can assist to gain comprehensive insight into a complexity of signal network of cellular proteins, leading to the identification of drug targets. Now it is a golden opportunity to employ the systems biology approach to unveil the global signal network of traditional medicine, which can explain its mode of action and pharmacology.

Acute lymphocytic leukemia (ALL) is the most common cancer of childhood. Its incidence rate is 1.6 per 100,000 men and women per year in United States. Around 15% of acute lymphocytic leukemia cases are T-cell acute lymphocytic leukemia (T-ALL), a disease caused by malignant transformation of T cells [3]. T-ALL pathogenesis is related to genetic alterations or aberrant expression of oncogenes or tumor suppressor genes. Though treatment outcomes for T-ALL have been much improved, novel lead compounds for T-ALL are necessary. Jurkat cells, which were established from a T-ALL patient, are a physiologically relevant tumor model of T-ALL [4].

Plant extracts or compounds are considered to be of great potential as therapeutic agents that can prevent or treat human cancers, immune disorders, and others.* Bidens pilosa* (Asteraceae), an edible herb, has been claimed to treat 41 diseases worldwide [5].* B. pilosa* has a complex phytochemistry. Naturally occurring polyynes have been shown to have a broad range of medicinal and biological properties such as antitumor activities [6, 7]. Of note, a group of polyynes were identified from* B. pilosa* [5]. Our group first demonstrated that some of them could promote apoptosis of endothelial cells and angiogenesis [8, 9]. Nevertheless, their antitumor effect and mechanism are poorly understood.

To gain a comprehensive picture of anticancer action of the polyynes present in* B. pilosa*, an integrative approach to use proteomics and bioinformatics methods was simultaneously applied in this study. We used the Jurkat cells as a model system to investigate the signal network of cellular proteins in responses to GHTT treatment. Biochemical methods were applied to verify the putative pathways of the responsive proteins of the phytocompound.

## 2. Material and Methods

### 2.1. Chemicals and Reagents

Most of the chemical reagents were purchased from Sigma-Aldrich (Saint Louis, MO, USA). Immobilized pH gradient (IPG) strips, buffers, and electrophoresis apparatus (Multiphor II and Protean IEF cell) were from Bio-Rad Laboratories (Hercules, CA, USA) and Amersham Biosciences (Denmark). SYPRO Ruby Protein Gel Stain was from Molecular Probes (Eugene, OR, USA). Trypsin (sequencing grade, modified) was from Promega (Madison, WI, USA). All other chemicals and solvents used in this study were of reagent grade or HPLC grade. GHTT was isolated from* B. pilosa* and structurally elucidated as published [10]. Briefly, the whole fresh plant was crushed and mixed with 10-fold volumes (L/kg) of 70% ethanol at room temperature. This crude extract was subsequently partitioned with ethyl acetate (EA) and* n*-butanol (BuOH), each with the same volume of water for 3 repeats. GHTT was purified from the BuOH fraction and used for this study. Antibodies against PARK7, VDAC2, LMNB1, NDUA5, and PRDX3 were purchased from Cell Signaling and/or Proteintech.

### 2.2. Cell Culture

Human T-ALL cell line, Jurkat cells (E6-1 clone, TIB 152), was obtained from American Type Culture Collection and cultivated in RPMI 1640 medium supplemented with 10% (v/v) fetal bovine serum, 10 mM HEPES, 1 mM pyruvate, 10 *μ*M *β*-mercaptoethanol, and 1% (v/v) penicillin-streptomycin glutamate at 37°C in a humidified incubator at 5% CO_2_. GHTT at 15 *μ*g/mL was added to the culture at cell density of 3–10 × 10^5^ cells/mL. The treatment was continued for 24 h before cell harvest by centrifugation at 624 g for 5 min. Cell pellets were washed twice in PBS and stored at −70°C until use for protein extraction.

### 2.3. Protein Extraction

Jurkat cells (~5 × 10^7^ cells) were lysed in 200 *μ*L of lysis buffer (7 M urea, 2 M thiourea, 4% 3-[(3-cholamidopropyl)dimethylammonio]-1-propanesulfonate (CHAPS), 1% dithiothreitol (DTT), 10 mM spermine, and 0.5% pharmalyte 3–10) for 1 h at room temperature with gentle vortex [11]. The viscous lysate was centrifuged at 100,000 g for 1 h to pellet the nucleic acids. The supernatant was recovered and stored in a deep freezer (−70°C) before use. Protein concentration was determined using* RC DC* protein assay reagent (Bio-Rad Laboratories, Hercules, CA).

### 2.4. Two-Dimensional Gel Electrophoresis (2DE)

Protein lysates from DMSO- and GHTT-treated Jurkat cells were first separated on an isoelectric focusing gel with pH ranges from pH 4 to 7, 5 to 8, or 6 to 9 and then a SDS-PAGE (18 × 20 cm) as a 2-dimensional gel with 10–16% acrylamide gradient. Protein on those gels was visualized by silver or fluorescence staining and scanned for image analysis using PDQuest 2D gel analysis software. Isoelectric focusing (IEF) of IPG strips (18 cm, pH 4–7 and pH 6–9) on Multiphor II and IPG strips (17 cm, pH 5–8) on Protean IEF Cell was essentially followed by the protocol of Gorg et al. (Electrophoresis, 2000, 21, 1037). Briefly, IPG dry strips were rehydrated for 16 h with protein (0.6–1 mg) in a rehydration solution containing 7 M urea, 2 M thiourea, 4% CHAPS, 1% DTT, 0.005% (v/v) bromophenol blue, 2% (v/v) IPG buffer for IPG pH 4–7 and pH 5–8 strips on Multiphor II, or 0.13% (w/v) Biolyte 5–8 and 0.07% (w/v) Biolyte 8–10 for IPG pH 5–8 strips on PROTEAN IEF Cell. IEF on Multiphor II system was conducted at 20°C for 74.6 Vh with voltages setting as follows: 300 V for 3 h, 300–1400 V linear gradient for 6 h, 1400 V for 10 h, 1400–3500 V linear gradient for 3 h, and 3500 V for 14 h. IEF on PROTEAN IEF Cell was conducted at 20°C for 92.7 Vh with voltages setting as follows: 300 V for 3 h, 300–1500 V linear gradient for 4 h, 1500 V for 8 h, 1500–4000 V for 3 h, 4000 V for 4 h, 4000–8000 V for 2 h, and 8000 V for 5 h. The strips were then stored at −70°C or equilibrated in 10 mL of 50 mM Tris-HCl, pH 8.8, 6 M urea, 30% (v/v) glycerol, 2% (w/v) SDS, trace bromophenol blue, and 65 mM DTT for 20 min. Alkylation was followed in 10 mL of the same buffer containing 135 mM iodoacetamide (IAA) instead of DTT for 20 min. The IPG strips were embedded with 0.5% w/v melted agarose on the top of 10–16% T gradient SDS-PAGE slabs (18 cm × 20 cm × 1 mm). The SDS-PAGE was run at 15°C with constant current setting at 12 mA/gel for 30 min and then at 24 mA/gel through the end of run. After electrophoresis, the gels were stained with SYPRO Ruby protein gel stain (Molecular Probes, Eugene, OR, USA) or silver staining with ammoniacal silver as described [11]. SYPRO Ruby stained gels were scanned at 100 dpi on a scanner (Typhoon 9200, Amersham Biosciences) and image of protein spots analysis was performed with the PDQuest 2D software (Bio-Rad).

### 2.5. Protein Identification by Mass Spectrometry (MS) and Database Searching

By gel-to-gel matching, the overestimating qualitative variations were removed and the spots constantly present in all gels from the same sample were further quantified and analyzed. Relative comparisons of protein spot volumes in fluorescence stained gels were analyzed using PDQuest 2D gel analysis software (Bio-Rad). Differentially expressed protein spots were then identified by in-gel trypsin digestion and analysis using MALDI-MS/MS or capillary LC ESI-MS/MS. Namely, the protein spots detected on 2DE gel were manually excised from the gel and cut into pieces for in-gel trypsin digestion. The gel pieces were dehydrated with acetonitrile for 10 min, vacuum dried, rehydrated with 55 mM DTT in 25 mM ammonium bicarbonate, pH 8.5, at 37°C for 1 h, and subsequently alkylated with 100 mM IAA in 25 mM ammonium bicarbonate, pH 8.5, at room temperature for 1 h. The pieces were then washed twice with 50% acetonitrile in 25 mM ammonium bicarbonate, pH 8.5 for 15 min each time, dehydrated with acetonitrile for 10 min, dried, and rehydrated with 25 ng trypsin (Promega, Madison, WI, USA) in 25 mM ammonium bicarbonate, pH 8.5, at 37°C for 16 h. Following digestion, tryptic peptides were extracted twice with 50% acetonitrile containing 5% formic acid for 15 min each time with moderate sonication. The extracted solutions were pooled and evaporated to dryness under vacuum.

Trypsin-digested hydrolysates from protein spots were subjected to MALDI MS and CID MS/MS analyses for protein identification using Q-Tof Ultima MALDI mass spectrometer (Waters/Micromass, Manchester, UK). Selected spots were also submitted to nano-LC-MS/MS analysis on a separate Q-Tof Ultima MS instrument equipped with a capillary LC system and a nano-ESI source for complementary protein identification.

For MALDI MS and MS/MS analysis, samples were premixed 1 : 1 with matrix solution (5 mg/mL CHCA, 2% ammonium citrate, and 0.1% TFA in 50% acetonitrile) and spotted onto a 96-well MALDI sample stage. Data dependent acquisition on the Q-TOF Ultima MALDI instrument was operated with predefined probe motion pattern and peak intensity threshold for switching over from MS survey scan to MS/MS. Precursor ions meeting the predefined criteria (*m/z* 800–3000 range with intensity above 10 counts) were selected for CID MS/MS using argon as collision gas and a mass dependent ±5 V rolling collision energy starting from the most intense peak. The quadrupole selection window for a precursor ion was set at 4 Da wide. The instrument was externally calibrated to 5 ppm accuracy over the mass range of* m/z* 800–3000 using a sodium iodide and PEG 200, 600, 1000, and 2000 mixtures, and Glu-Fibrinopeptide B was used as the lock mass calibrant during data processing.

For LC-MS/MS analysis on the nano-LC-Q-Tof Ultima MS system, tryptic peptide samples were first injected into an autosampler, trapped, desalted on a precolumn (LC-Packings PepMap C18 *μ*-Precolumn Cartidge, 300 *μ*m I.D. × 5 mm, 5 *μ*m; Dionex), and separated on an analytical C18 capillary column (Zorbax 300 SB C18, 75 *μ*m I.D. × 15 cm, 5 *μ*m, Micro-Tech Scientific) connected online to the mass spectrometer at 300 nL/min flow rate using a 40 min gradient of 5% to 40% acetonitrile in 0.1% formic acid. For routine protein identification analysis, the 1 s survey scans were acquired over the mass range* m/z* 400–2000 and a maximum of 3 concurrent. Data dependent MS/MS acquisitions were triggered for 2+, 3+, and 4+ charged precursors.

After data acquisition, MS and MS/MS data acquired on MALDI- and LC-MS runs were processed into peak list files (.txt and .pkl) using the Micromass ProteinLynx Global Server (PGS) 2.0 data processing software. The peak list files were used for peptide/protein identification based on peptide mass fingerprinting and/or MS/MS ions searching using the online Mascot database search engine (http://www.matrixscience.com/) against the SwissProt* Homo sapiens* protein sequence database with the following parameters: peptide mass tolerance, 50 ppm; MS/MS ion mass tolerance, 0.25 Da; missed cleavage allowed, 1; variable modifications, methionine oxidation; and fixed modifications, cysteine carbamidomethylation. Only significant peptide hits as defined by Mascot significant threshold (*p* < 0.05) were considered initially. In addition, a minimum total score of 20 comprising at least one peptide match of ion score more than 20 was arbitrarily set as the threshold for acceptance.

### 2.6. Flow Cytometry

To measure the mitochondrial membrane potential, Jurkat cells were preloaded with tetramethylrhodamine methyl ester (TMRM, 10 nM) at 37°C in the dark for 30 min. After washing, the cells were incubated with DMSO vehicle or GHTT at the indicated doses for an additional 30 min. The cells underwent BD LSR II flow cytometry analysis. The data were processed using FCS express 3 software (De Novo Inc., CA, USA). For cell apoptosis, Jurkat cells were incubated with DMSO vehicle or GHTT at the indicated doses for 30 min. After washing, the cells were stained with annexin V plus propidium iodide (PI) and, in turn, underwent flow cytometry analysis and FCS express 3 analysis.

### 2.7. Ingenuity Pathways Analysis

Using a web-based entry tool developed by Ingenuity systems [12], in which specific molecular network of direct physical, transcriptional, and enzymatic interactions could be observed between mammalian orthologs. For better understanding the biological meaning of changes in protein expression in Jurkat cells treated with DMSO and GHTT, we constructed possible candidate signaling pathways of 22 of their responsive proteins, identified by 2DE/MS analyses, in Table 1 by using the Ingenuity software.

### 2.8. Statistics

Data from three or more independent experiments are presented as mean ± standard error (SE). Comparisons between multiple groups were made with ANOVA. *p* < 0.05 was considered significant.

## 3. Results and Discussion

### 3.1. Identification of the Differentially Expressed Proteins in Jurkat Cells in Response to GHTT Using Two-Dimensional Gel Electrophoresis Coupled to Mass Spectroscopy (2DE/MS)

**Figure 1:**
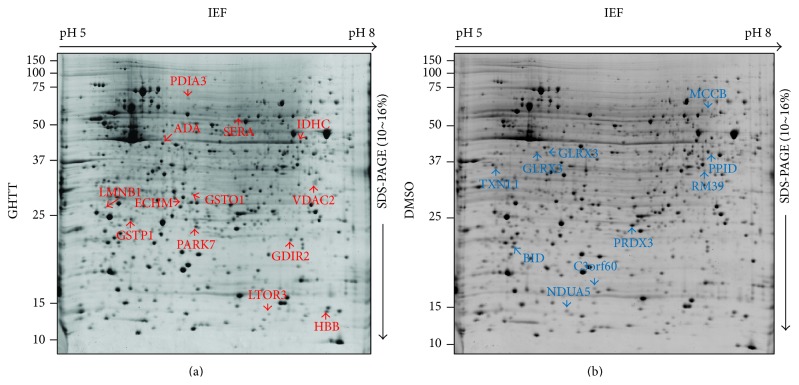
2DE gels of cellular proteins from the GHTT-treated (a) and DMSO-treated Jurkat T cells (b). Upregulated proteins (red on gel (a)) and downregulated proteins (blue on gel (b)) were identified by MS or MS/MS analysis. Identity of these spots is listed in Table 1.

**Figure 2:**
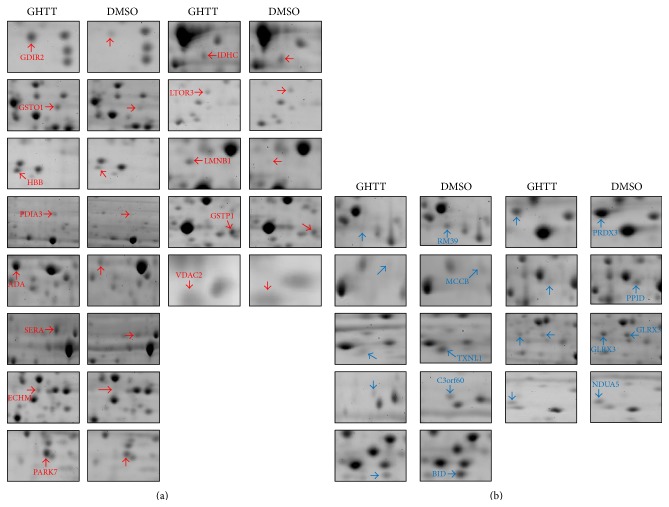
Enlarged view of the spots up- and downregulated by treatment with DMSO and GHTT. Red and blue circles indicate the increase fold and decrease fold of the proteins listed in Table 1, respectively.

To explore novel signal pathway network of the phytocompound, GHTT, on Jurkat cells, the 2DE/MS was engaged to compare the protein expression profiles of Jurkat cells treated with DMSO vehicle and GHTT. With this technology, we were able to routinely obtain representative, high resolution, and highly reproducible 2D protein profiles of Jurkat cells (Figure 1). Around 1,200–2,000 protein spots with a molecular mass range of 10–150 kDa in each gel were detected (Figure 1). In order to avoid erroneous and ambiguous evaluation of the experimental data, comparative difference of the protein spots was verified at least in three replicates from each batch of T cells collection. Red and blue circles denoted the up- and downregulation of the proteins in GHTT-treated Jurkat cells as opposed to those in control cells (Figure 1). Differentially expressed proteins of 22 spots showing >1.5-fold change were further identified. Table 1 lists proteins identified by MALDI-MS/MS or capillary LC-ESI-MS/MS and databases search based on peptide mass fingerprinting and MS/MS ions search using Mascot search program. Thirteen upregulated and nine downregulated proteins could be identified (Figure 2). In fact, some of the protein spots could not be identified due to low protein abundance or lack of peptides digestible with trypsin (data not shown).

Thirteen proteins, including Rho GDP-dissociation inhibitor 2 (GDIR2), glutathione transferase omega 1 (GSTO1), hemoglobin beta chain (HBB), protein disulfide isomerase A3 (PDIA3), adenosine deaminase (ADA), D-3-phosphoglycerate dehydrogenase (SERA), DJ-1 protein (PARK7), cytoplasmic NADP-dependent isocitrate dehydrogenase (IDHC), mitogen-activated protein kinase kinase 1 interacting protein 1 (LTOR3), LMNB1 protein (LMNB1), glutathione S-transferase P (GSTP1), and anion-selective channel protein 2 (VDAC2), were found to be upregulated in GHTT-treated Jurkat cells compared to control Jurkat cells (red, Figures 1 and 2). Nine proteins, mitochondrial 39S ribosomal protein L39 (RM39), methylcrotonoyl-CoA carboxylase beta chain (MCCB), thioredoxin-like protein 1 (TXNL1), chromosome 3 open reading frame 60 (C3orf60), BH3 interacting domain death agonist (BID), thioredoxin-dependent peroxide reductase, mitochondrial precursor (PRDX3), 40 kDa peptidyl-prolyl cis-trans isomerase (PPID), thioredoxin-like protein 2 (GLRX3), and NADH-ubiquinone oxidoreductase 13 kDa-B subunit (NDUA5), were observed to be downregulated in GHTT-treated Jurkat cells compared to control Jurkat cells (blue, Figures 1 and 2).

**Figure 3:**
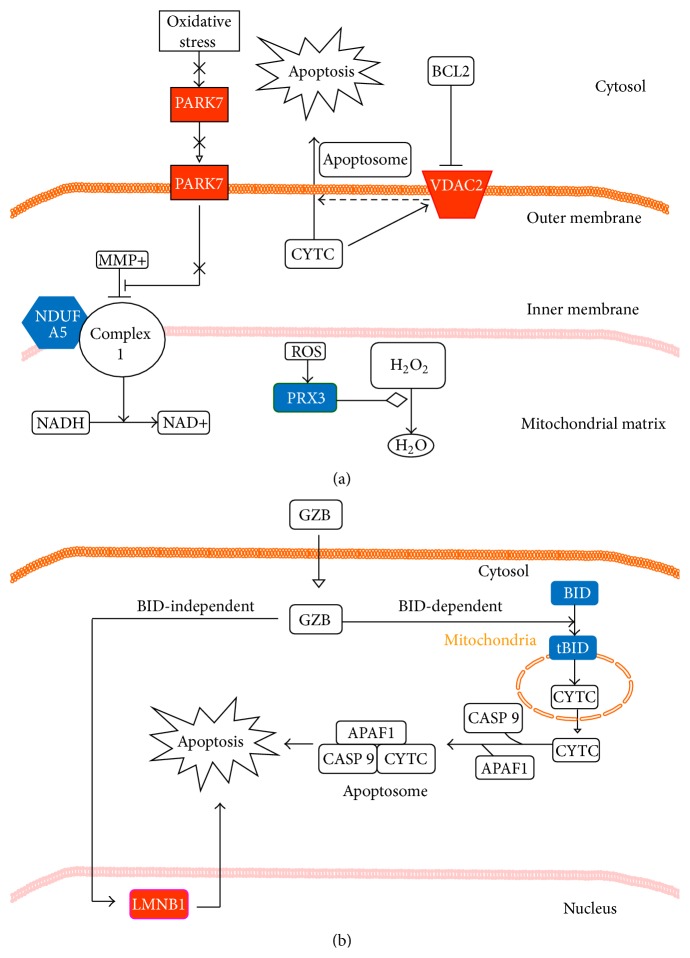
Pathways analysis of the proteins listed in Table 1 by means of the Ingenuity software. Mitochondrial dysfunction pathway (a) and granzyme B (GZB) signaling pathway (b) are proposed according to the structured network knowledge-based strategy. In (a), increase in the permeability of VDVC proteins in the outer mitochondrial membrane is assumed to allow for CYTC and, therefore, relevant to mitochondrial dysfunction and apoptosis. The VDVC-mediated apoptosis involves the formation of apoptosome and an activation of the caspase cascade. PRDX3 acts as an antioxidant protein to catalyze the degradation/reduction of hydrogen peroxide to water. Oxidative stress promotes the formation of ROS in mitochondrial complexes 1 to 4 and can cause mitochondrial damage. PARK7 acts as an antioxidant player and antagonizes the loss of mitochondrial function. In (b), GZB exerts its apoptotic function via the BID-dependent and BID-independent pathways. In BID-dependent route, GZB degrades BID to generate its active truncated BID (tBID), thereby inducing cell death via the formation of apoptosome and activation of caspases. In BID-independent route, GZB can activate different caspases and caspase substrates (e.g., LMNB1, etc.) independent of BID cleavage, leading to apoptosis. Red and blue indicate the proteins which are up- or downregulated by the plant compound, GHTT, in Jurkat cells. Translocation (—▹), activation (⟶), inhibition (⊣), and catalysis (—⋄) are indicated.

To identify specific signaling pathways in response to GHTT treatment in Jurkat cells, we analyzed the 22 up- and downregulated proteins using the Ingenuity software similar to the publications [12, 13]. Two likely pathways related to mitochondrial function (Figure 3(a)) and cell survival (Figure 3(b)) in Jurkat cells were postulated to elucidate the pharmacological action and mechanism of GHTT. The first pathway was relevant to mitochondrial function, including the participation of PRDX3, VDAC2, and PARK7 proteins, with an expression change over 1.5-fold, as shown in Figure 3(a). PRDX3 is multifunctional protein in mitochondria, involving the regulation of ROS accumulation and apoptosis [10]. PRDX3 acted as a mitochondrial ROS scavenger and, therefore, diminished ROS content under cellular oxidative conditions [14, 15]. However, PRDX3 was associated with carcinogenesis in Caucasian patients [16]. VDAC2 was also shown to regulate apoptosis. During apoptosis, the permeability of VDAC2 was increased and, in turn, led to the release of cytochrome c (CYTC) [17]. PARK7, a DJ-1 oncogene, was thought to maintain mitochondrial function during oxidative stress and thereby alter mitochondrial dynamics and autophagy indirectly [18]. In this work, the protein expression level of PRDX3 was decreased (−3.6-fold) in Jurkat cells by GHTT. In parallel, the protein level of VDAC2 (+1.5-fold) was increased by GHTT in the cells (Figure 3(a)). Both data suggest that GHTT treatment promotes the dysfunction of mitochondria and apoptotic activity in Jurkat cells. However, the protein level of PARK7 (+1.6-fold) was increased after GHTT treatment, suggesting that the ability of PARK7 to maintain mitochondrial function was induced by GHTT in an attempt to balance the mitochondria dysfunction.

The second likely pathway was related to cell death, involving the participation of BID and LMNB1. Granzyme B (GZB) was reported to promote apoptosis through two main pathways, either through BID-dependent CYTC release or through direct caspase processing and activation [19]. Further, GZB also directly cleaves several caspase substrates such as LMNB1 and others via BID-independent death pathway [20]. In this study, the protein expression level of BID was decreased (−3.8-fold). However, tBID was not detected in this study. In contrast, the protein expression level of LMNB1 was significantly increased (+5.1-fold) in Jurkat cells after GHTT treatment (Figure 3(b)). These data suggest that GZB-mediated cell death, which was implicated in LMNB1 but not BID, was activated by GHTT in Jurkat cells (Figure 3(b)). For the rest of GHTT-responsive proteins, the Ingenuity software failed to predict any matched biological pathways. However, PDIA3 [21], TXNL1 [22], and GLRX3 [23–25] were also proposed to be implicated in cancers. Upon the recognition of tumor antigen, GZB, released by cytotoxic T cells, can induce cell death in tumor. Since GHTT was involved in GZB-mediated tumor cell death, this raised the possibility that GHTT could potentiate GZB-mediated tumor cell death, which could be beneficial for tumor therapy.

Overall, the structured network knowledge-based strategy using the Ingenuity software pointed to two putative apoptotic pathways in Jurkat cells in response to the plant chemical, GHTT. This approach not only simplifies the processing of proteomics data (proteins and their expression change and signal network) but also provides constructive information about the signaling pathways and mechanism of GHTT. Of course, the putative signaling pathways need to be ascertained with further experiments.

### 3.2. Effect of GHTT on Mitochondrial Dysfunction and Apoptosis of Jurkat Cells

**Figure 4:**
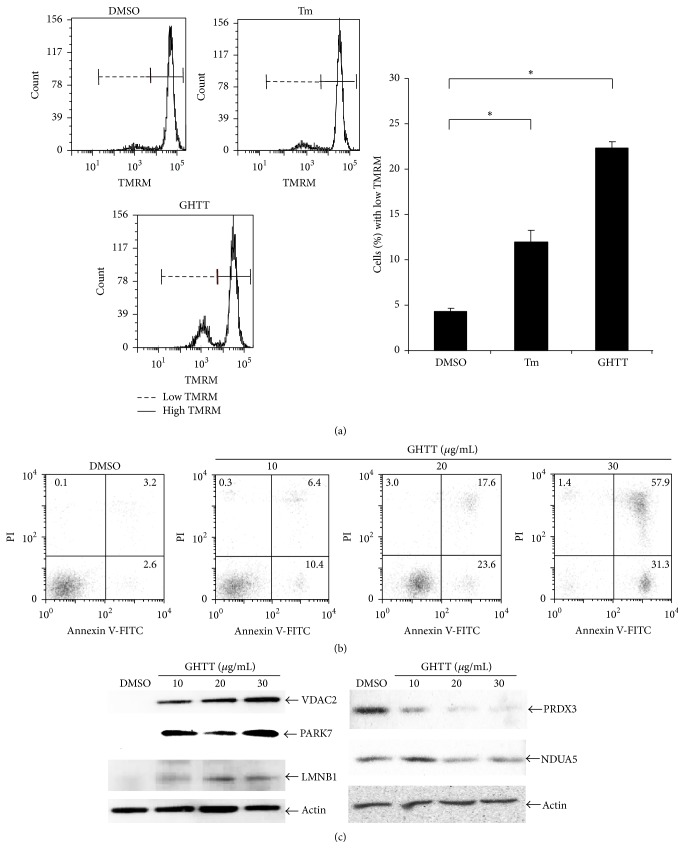
Effect of GHTT on mitochondrial membrane potential (a) and apoptosis (b) in Jurkat cells. (a) Jurkat cells were incubated with TMRM. After washing, the cells were treated with GHTT, DMSO, a negative control, and tunicamycin (Tm, 10 *μ*g/mL), a positive control, followed by flow cytometry analysis. (b) Jurkat cells were incubated with GHTT and DMSO. After washing, the cells were stained with PI and annexin V and subjected to flow cytometry analysis. (c) Total lysates of Jurkat cells treated with GHTT and DMSO underwent SDS-PAGE. Following protein transfer, the membrane was blotted with the indicated antibodies.

Next, we wanted to verify the effect of GHTT on mitochondrial function and cell survival of Jurkat cells. TMRM has been used as a fluorescent dye to detect mitochondrial membrane potential, an indication of mitochondrial function [26]. As shown in Figure 4(a), less than 5% of Jurkat cells decreased their mitochondrial membrane potential in exposure to DMSO vehicle (DMSO, Figure 4(a)). In contrast, 12% of the cells decreased their mitochondrial membrane potential in exposure to tunicamycin, an ER stress inducer (Tm, Figure 4(a)). Further, 23% of the cells decreased their mitochondrial membrane potential in exposure to GHTT (GHTT, Figure 4(a)). The results corroborate the function of GHTT in causing mitochondrial dysfunction, akin to the predictive data deduced from proteomics and bioinformatics analyses. In parallel, we tested the effect of GHTT on life and death of Jurkat cells. DMSO vehicle increased apoptosis by 5.8% (DMSO, Figure 4(b)). In contrast, GHTT elevated apoptosis from 16.8% to 88.2% (GHTT, Figure 4(b)). This elevation was dose-dependent. Besides, we verified the expression level of key proteins involved in mitochondrial dysfunction and cell death. Consistent with the proteomics data (Figure 2), VDAC2, PARK7, and LMNB1 were upregulated in Jurkat cells in response to GHTT (Figure 4(c)). In contrast, NDUFA5 and PRX3 were downregulated in Jurkat cells in response to GHTT (Figure 4(c)). The regulation of the above proteins in Jurkat cells by GHTT appeared to be dose-dependent. The data confirmed that mitochondrial dysfunction and cell death are related to these proteins.

Overall, the biochemical studies confirm the omics approach-driven discovery of signaling pathways in Jurkat cells and reveal a novel molecular basis of mitochondrial dysfunction and cell death of the anticancer compound, GHTT, in T-ALL cells. This concept-of-proof study exemplifies the feasibility of the novel interdisciplinary approach to combine proteomics and bioinformatics methods in basic research and application of herbal medicine and its active components.

## Figures and Tables

**Table 1:** Differentially expressed proteins in GHTT-treated Jurkat T cells identified using 2DE/MS analysis.

ID	Protein name	Accession number	Theor. Mr (kDa)	Theor. pI	Sequence coverage (%)	Number of peptides matched	Avg. of fold difference^a^	Functional categorization^b^	Identification method
GDIR2	Rho GDP-dissociation inhibitor 2	P52566	22974	5.10	41	5	+4.0	Signal transduction	MS, MS/MS

GSTO1	Glutathione S-transferase omega 1	P78417	27548	6.23	44	12	+2.0	Detoxification	MS, MS/MS, ESI-MS/MS

HBB	Hemoglobin beta chain	P68871	15871	7.26	15	4	+3.4	Transport	MS/MS

PDIA3	Protein disulfide isomerase A3 precursor	P30101	56747	5.98	28	15	++	Metabolism	MS/MS

ADA	Adenosine deaminase	P00813	40555	5.54	35	14	+3.2	Metabolism; energy pathways	MS, MS/MS

SERA	D-3-phosphoglycerate dehydrogenase	O43175	56483	6.31	23	10	+2.3	Metabolism; energy pathways	MS, MS/MS

ECHM	Enoyl-CoA hydratase, mitochondrial precursor	P30084	31379	8.34	33	7	+2.9	Metabolism; energy pathways	MS, MS/MS

PARK7	DJ-1 protein (oncogene DJ1)	Q99497	19878	6.33	42	7	+1.6	Nucleoside, nucleotide, and nucleic acid metabolism	MS, MS/MS

IDHC	Isocitrate dehydrogenase [NADP] cytoplasmic	O75874	46630	6.53	28	9	+1.7	Metabolism; energy pathways	MS, MS/MS

LTOR3	Mitogen-activated protein kinase kinase 1 interacting protein 1	Q9UHA4	13614	6.73	85	8	+1.5	Signal transduction	MS, MS/MS

LMNB1	LMNB1 protein	AAH78178	38118	5.37	26	9	+5.1	Miscellaneous	MS, MS/MS

GSTP1	Glutathione S-transferase P	P09211	23210	5.44	40	6	+1.8	Metabolism; energy pathways	MS, MS/MS, ESI-MS/MS

VDAC2	Voltage-dependent anion-selective channel protein 2	P45880	38069	6.32	9	7	+1.5	Transport	MS, MS/MS

RM39	Mitochondrial 39S ribosomal protein L39	Q9NYK5	34204	6.47	26	7	—	Metabolism	MS, MS/MS

MCCB	Methylcrotonoyl-CoA carboxylase beta chain	Q9HCC0	61294	7.57	31	12	—	Metabolism; energy pathways	MS, MS/MS

TXNL1	Thioredoxin-like protein 1	O43396	32100	4.84	58	10	−4.4	Metabolism	MS, MS/MS

C3orf60	Chromosome 3 open reading fragment 60	AAH02873	20337	8.48	29	5	−2.1	Miscellaneous	MS, MS/MS

BID	BH3 interacting domain death agonist	P55957	21981	5.27	41	7	−3.8	Apoptosis	MS, MS/MS

PRDX3	Thioredoxin-dependent peroxide reductase, mitochondrial precursor	P30048	27675	7.67	14	3	−3.6	Metabolism; energy pathways	MS/MS

PPID	40 kDa peptidyl-prolyl cis-trans isomerase	Q08752	40607	6.76	36	12	−2.0	Metabolism; energy pathways	MS, MS/MS, ESI-MS/MS

GLRX3	Thioredoxin-like protein 2	O76003	37408	5.31	34	13	−2.7	Metabolism	MS, MS/MS, ESI-MS/MS

GLRX3	Thioredoxin-like protein 2	O76003	37408	5.31	31	10	−3.5	Metabolism	MS, MS/MS, ESI-MS/MS

NDUA5	NADH-ubiquinone oxidoreductase 13 kDa-B subunit	Q16718	13319	5.76	49	7	−2.1	Metabolism; energy pathways	MS/MS

^a^Data were obtained from the analysis of protein spots (GHTT versus control) using PDQuest software. Symbol — represents that the respective protein spot in the GHTT gel was not detected.

^b^Proteins were queried in Human Protein Reference Database (http://www.hprd.org/) for their functional categorization.
